# A case of Hirschsprung’s disease with segmental dilatation of the colon

**DOI:** 10.1186/s40792-023-01602-1

**Published:** 2023-02-15

**Authors:** Hideyuki Yokokawa, Mariko Yoshida, Shinya Takazawa, Mai Kutsukake, Kanako Omata, Shoko Ogawa, Jun Fujishiro

**Affiliations:** Department of Pediatric Surgery, The University of Tokyo Hospital, 7-3-1 Hongo, Bunkyo-Ku, Tokyo, 113-8655 Japan

**Keywords:** Segmental dilatation of the intestine, Segmental dilatation of the colon, Hirschsprung’s disease

## Abstract

**Background:**

Segmental dilatation of the colon (SDC) is a rare disease that is characterized by an abrupt segment of dilated colon between regions of normal-sized colon. We herein report a case of SDC associated with Hirschsprung’s disease (HD).

**Case presentation:**

The patient developed abdominal distension soon after birth, and enema examination showed localized intestinal dilatation from the descending colon to the sigmoid colon with significant caliber changes on both the oral and anal sides of the dilated colon. The findings of the rectal mucosal biopsy were consistent with HD. We considered this case to be a combination of HD and SDC and performed laparoscopic-assisted Soave pull-through with resection of the dilated colon when the patient was 7 months old. Resected specimens showed steep caliber changes on the oral and anal sides of the dilated colon. In the pathological examination, no ganglion cells were found in the submucosa on the anal side of the dilated colon. Based on the above findings, we finally made the diagnosis of HD with SDC.

**Conclusion:**

In HD with a characteristic dilated colon, the possibility of SDC should be considered.

## Introduction

Segmental dilatation of the intestine (SDI) is a rare condition in which there is an abrupt segment of dilated intestine between regions of normal intestine without obstruction. Among SDI cases, those with lesions in the colon are called segmental dilatation of the colon (SDC) [1, 2]. SDC differs from Hirschsprung’s disease (HD) in that the histological examination shows the presence of normal ganglionic cells of the gastrointestinal autonomous plexus [1]. We herein report a case of SDC associated with HD. To the best of our knowledge, this is the first case of HD with SDC reported in the English literature.

## Case presentation

**Fig. 1:**
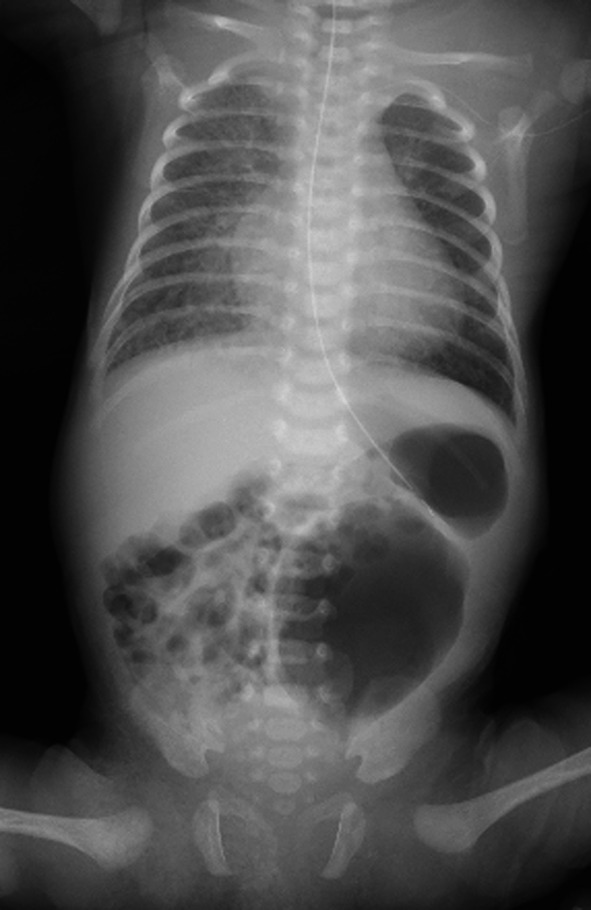
Abdominal X-ray 1 day after birth. The abdominal X-ray showed a dilated intestine in the left lower abdomen

**Fig. 2:**
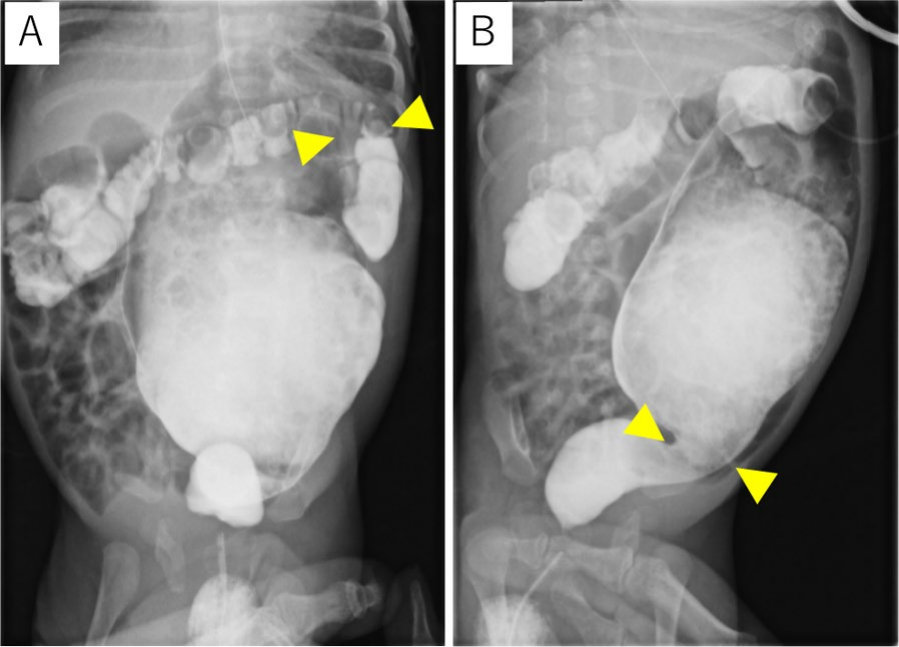
The contrast enema on Day 41. The constant enema showed localized intestinal dilatation from the descending colon to the sigmoid colon with abrupt caliber changes (arrowheads) in the oral and anal sides of the dilated colon. **A** Spine position and **B** left lateral position

**Fig. 3:**
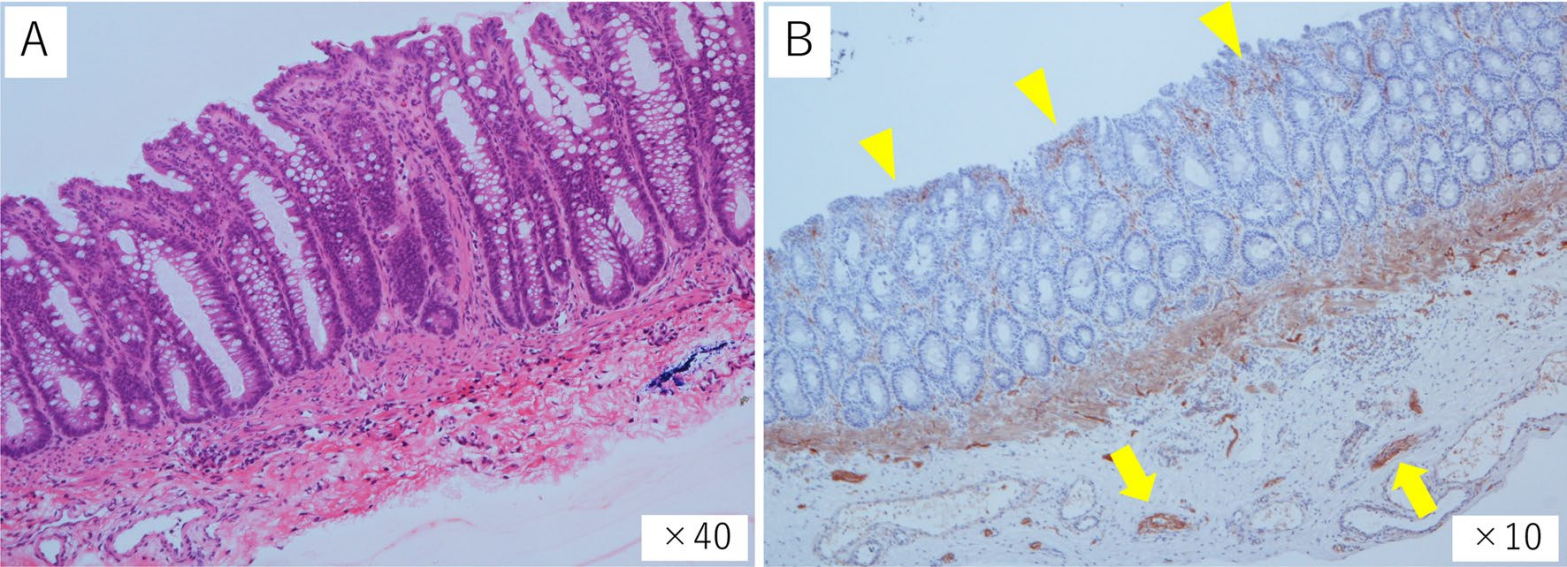
Histological findings of the rectal mucosal biopsy on Day 63. Histology of the rectal mucosal biopsy showed **A** no ganglion cells in the rectal submucosa (hematoxylin and eosin) and **B** increased acetylcholinesterase-positive nerve fibers in the lamina propria (arrowheads) and immediately under the muscularis mucosa (arrows)

A female baby was born in another hospital by emergency cesarian section at 35 weeks and 6 days with a birth weight of 1802 g. She was a dichorionic diamniotic twin and diagnosed with trisomy 21, ventricular septal defect (VSD), atrial septal defect, and patent ductus arteriosus (PDA) after birth. Abdominal distention was noted soon after birth. An enema was administered without waiting for meconium excretion. On Day 1, a dilated colon in the left lower abdomen on abdominal X-ray (Fig. 1) was observed, but defecation was well controlled with enemas. She was transferred to our hospital on Day 3 for cardiopulmonary control and further evaluation of the dilated colon. Pulmonary artery banding and PDA clipping were performed on Day 22. The contrast enema at 41 days after birth showed localized intestinal dilatation from the descending colon to the sigmoid colon, and the oral and anal sides of the dilatation were steeply shifted to the normal-sized colon (Fig. 2A, B). A rectal mucosal biopsy 63 days after birth showed increased acetylcholinesterase-positive nerve fibers in the rectal mucosa and submucosa and no ganglion cells in the submucosa (Fig. 3A, B), which was consistent with HD. As steep caliber changes on the oral and anal sides of the dilated colon (Fig. 2A, B) are characteristic findings in SDC, she was diagnosed with the combination of HD and SDC.

**Fig. 4:**
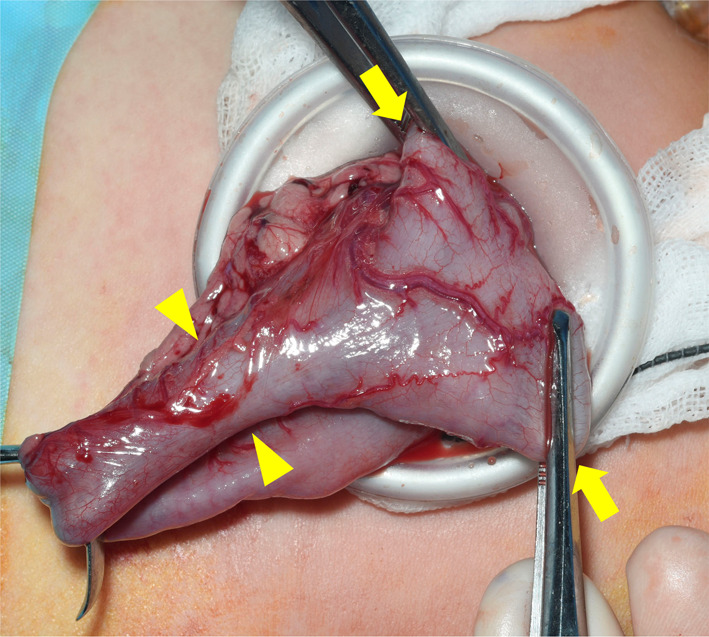
Intraoperative findings. The normal-sized transverse colon (arrowheads) on the oral side of the dilated colon (arrows) was elevated from the umbilical incision and separated

The patient underwent laparoscopic-assisted Soave pull-through with resection of the dilated colon at the age of 7 months. Her body weight was 5447 g. On laparoscopy, the dilated descending and sigmoid colon and upper rectum were identified, and the normal-sized transverse colon on its oral side was elevated from the umbilical incision and separated (Fig. 4). The frozen section diagnosis of the normal-sized transverse colon on the oral side of the dilation revealed normal ganglion cells in the intermuscular and submucosal plexus. The affected bowel was pulled out through the umbilical incision because it was difficult to remove it from the abdominal cavity through the anus. Soave pull-through and coloanal anastomosis were performed.

**Fig. 5:**
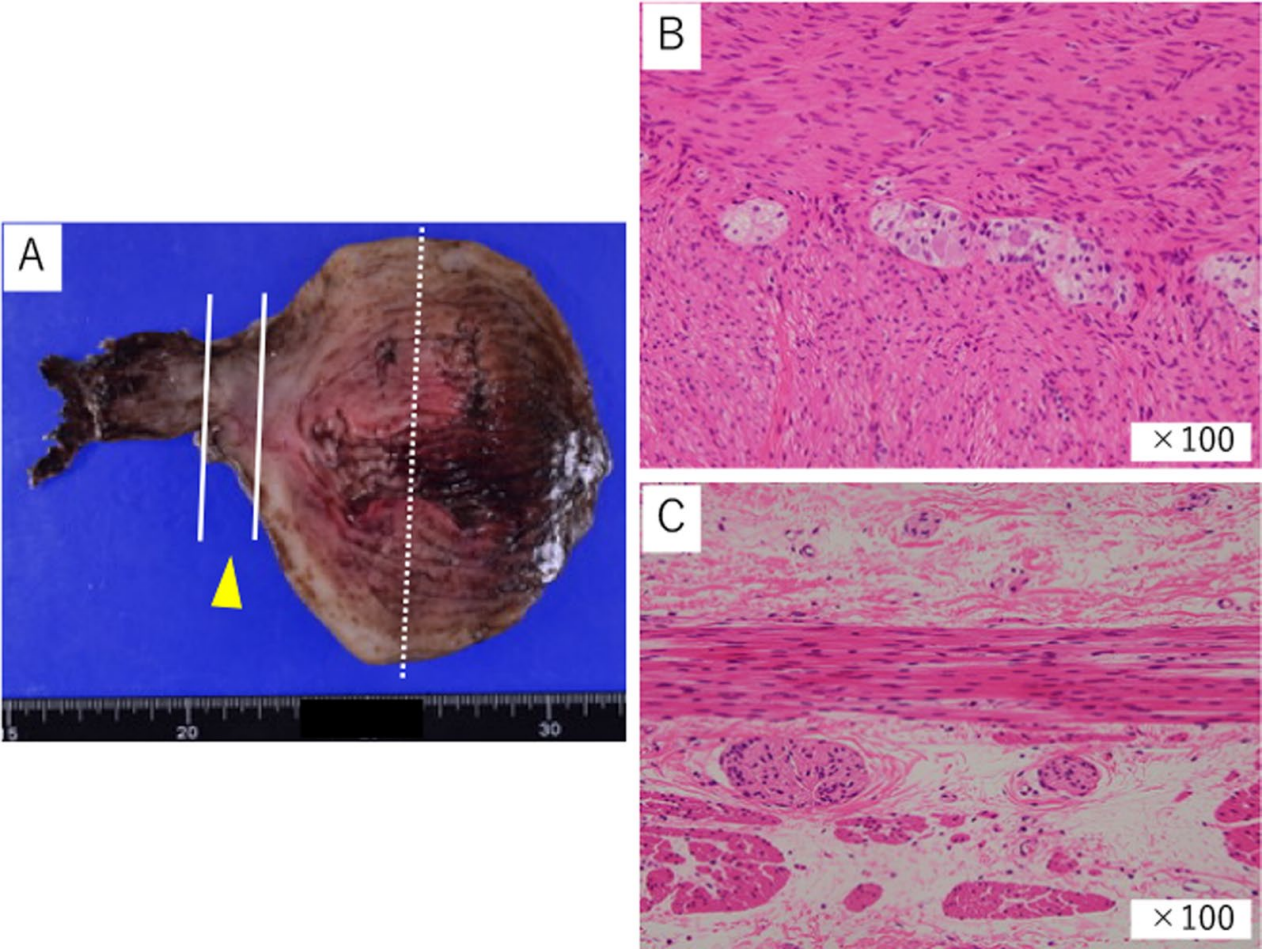
Histological findings of the resection specimens. **A** Resection specimens showed steep caliber changes in the bowel diameter on both the oral and the anal sides of the dilated bowel. **B** Normal ganglion cells were seen circumferentially within the submucosa and the muscularis propria in the dilated colon 5 cm from the oral edge (dotted line in **A**). **C** At 9–11 cm from the oral edge (between the solid lines in **A**), there was a decrease in the number of ganglion cells in the muscularis propria, corresponding to the transition zone. **C** Shows the area where no ganglion cells were observed

Resection specimens showed steep changes in bowel diameter on both sides of the dilated colon (Fig. 5A). The pathological examination revealed that the dilated colon and its oral side were ganglionic (Fig. 5A, B), and its anal side was aganglionic. The transition zone was located just on the anal side of the dilated colon (Fig. 5A, C). Muscle layer of the dilated colon was normal.

The postoperative course was uneventful, and the patient was discharged home on the 38th day after surgery. One year has passed since the surgery, and she still exhibits good defection control and sufficient weight gain.

## Discussion

SDI is an uncommon entity described for the first time by Swenson and Rathauser in 1959 [2]. The diagnostic criteria for SDI were proposed (Table 1), including no intrinsic or extrinsic barrier distal to the dilatation and a normality of the neuronal plexus [2, 3]. Sakaguchi et al. reported concurrent intestinal malformations in four of 28 cases [3]. There are some reports of concurrent congenital diseases, such as VSD, PDA and trisomy 21, as in our case [3–5]. There are some etiologic theories of SDI, but the exact pathogenesis of SDI is still unknown [3, 6], and the reason why SDI is concurrent with cardiovascular malformations and 21 trisomy is also unknown.

**Table 1:** The diagnostic criteria of segmental dilatation of the intestine

(a) Limited bowel dilatation with a three- to fourfold increase in size
(b) An abrupt transition between the dilated and normal bowel
(c) No intrinsic or extrinsic barrier distal to the dilatation
(d) A clinical picture of intestinal occlusion or subocclusion
(e) Normality of the neuronal plexus
(f) Complete recovery after resection of the affected segment

The diagnosis of definitive SDI occurred when all criteria were met, and possible SDI was diagnosed solely based on anatomical features [(a)–(c)] without occlusive findings, surgery or histological examination [2, 3]

It is important to differentiate SDC from HD because the dilated intestine is a common finding in both diseases. While the caliber change is observed in the anal side of the dilated intestine in both diseases, clear caliber changes should be observed on both oral and anal sides of the dilated segment in SDC [7]. In our case, there were clear caliber changes on both sides of the dilated colon. The rectal mucosal biopsy was consistent with HD, but the caliber change on the oral side of the dilated colon was not explained by HD only. Therefore, we considered the case to be SDC in combination with HD. By definition (Table 1), SDI has no stenosis on the distal side of the dilated intestine and a normal neuronal plexus. However, there have been some reports of SDI with anorectal malformations, which usually have stenosis or occlusion of the rectum [9]. Furthermore, contrary to the diagnostic criteria, various pathological findings, including abnormality of ganglion cells or distribution of the plexii of ganglion cells, have also been reported in SDI [3, 8]. Thus, the disease concept of SDI has not yet been established in a simple manner. It is appropriate to regard it as a group of diseases with localized intestinal dilatation [7]. Therefore, we considered this case to be SDC associated with HD.

In SDI patients, resection of the dilated segment will provide a good prognosis [3]. Meanwhile, the goal of treatment of HD is resection of the aganglionic intestine followed by bringing the normal ganglionic intestine down to the distal rectum close to the anus, usually without resection of the dilated intestine. In our case, we performed laparoscopic-assisted Soave pull-through with resection of the dilated colon because we thought the patient had HD with SDC. In a previously reported case of SDC with anorectal malformation, perineal anoplasty alone did not improve the dilated colon or constipation. The patient subsequently needed resection of the dilated colon [9]. There is also one Japanese case report describing a case of HD with SDC [10]. In the report, enema examination showed marked dilatation of the sigmoid colon. They performed rectal biopsy and colostomy at the oral side of the dilated sigmoid colon when the patient was 8 days of age. The findings of the rectal mucosal biopsy were consistent with HD. At 10 months of age, the dilated colon remained despite the colostomy at the oral side, and laparoscopic-assisted pull-through and resection of the dilated colon were performed under the diagnosis of HD with SDC. Unfortunately, it is difficult to discuss the etiological relationship of SDC and HD because the etiology of SDI is unknown. In the previous reports, it has not been discussed about that. Thus, in the presence of other organic diseases, it is important to suspect the coexistence of SDI in patients that have characteristic bowel dilatation.

## Conclusion

We herein report a rare case of SDC associated with HD, diagnosed due to the presence of steep caliber changes on the oral and anal sides of the dilated colon. We performed laparoscopic-assisted Soave pull-through with resection of the dilated colon and obtained a good prognosis. In HD with a segmentally dilated colon, the possibility of SDC should be considered. Further accumulation of knowledge on the pathogenesis of SDI is desirable.

## Data Availability

The patient’s data are not available because personal information should be protected.

## Abbreviations

HD: Hirschsprung’s disease
PDA: Patent ductus arteriosus
SDC: Segmental dilatation of the colon
SDI: Segmental dilatation of the intestine
VSD: Ventricular septal defect
